# Tuning the Permeability–Selectivity Trade-Off in Activated Carbon/PES Mixed Matrix Membranes via Compaction and Vapor-Induced Phase Separation

**DOI:** 10.3390/membranes16080254

**Published:** 2026-07-25

**Authors:** Asseghaf Bintang Ramadhani, Jason Nathanael Thionardo, Muhammad Mirza Rahardianto, Annas Zakky Firmansyah, Kartika Nur ‘Anisa’, Chandrawati Putri Wulandari, Muslim Mahardika, Yudan Whulanza, Ario Sunar Baskoro, Thanongsak Thepsonthi, Nor Hasrul Akhmal Ngadiman, Gunawan Setia Prihandana

**Affiliations:** 1 Department of Industrial Engineering, Faculty of Advanced Technology and Multidiscipline, Universitas Airlangga, Jl. Dr. Ir. H. Soekarno Kampus C, Mulyorejo, Kec. Mulyorejo, Surabaya 60115, Jawa Timur, Indonesia; asseghaf.bintang.ramadhani-2022@ftmm.unair.ac.id (A.B.R.); jason.nathanael.thionardo-2022@ftmm.unair.ac.id (J.N.T.); muhammad.mirza.rahardianto-2022@ftmm.unair.ac.id (M.M.R.); annas.zakky.firmansyah-2022@ftmm.unair.ac.id (A.Z.F.); kartika.nuranisa@ftmm.unair.ac.id (K.N.‘A.); chandrawati.p.w@ftmm.unair.ac.id (C.P.W.); 2 Research Group of Industrial Management, System Engineering & Manufacturing, Universitas Airlangga, Jl. Dr. Ir. H. Soekarno Kampus C, Mulyorejo, Kec. Mulyorejo, Surabaya 60115, Jawa Timur, Indonesia; 3 Department of Mechanical and Industrial Engineering, Faculty of Engineering, Universitas Gadjah Mada, Jalan Grafika No. 2, Yogyakarta 55281, Indonesia; muslim_mahardika@ugm.ac.id; 4 Department of Mechanical Engineering, Faculty of Engineering, Universitas Indonesia, Kampus UI, Depok 16425, Jawa Barat, Indonesia; yudan@eng.ui.ac.id (Y.W.); ario@eng.ui.ac.id (A.S.B.); 5 Department of Industrial Engineering, Faculty of Engineering, Burapha University, 169 Longhard Bangsaen Beach Rd., Saensook, Muang, Chonburi 20131, Thailand; thanongsak@eng.buu.ac.th; 6 Faculty of Mechanical Engineering, Universiti Teknologi Malaysia, Johor Bahru 81310, Johor, Malaysia; norhasrul@utm.my

**Keywords:** composite membrane, activated carbon, polyethersulfone, vapor-induced phase separation, good health

## Abstract

This study investigates the synergistic effects of compaction pressure and vapor-induced phase separation (VIPS) on the morphological, mechanical, and initial filtration properties of activated carbon/polyethersulfone composite block membranes. Membranes were fabricated using varying compaction pressures (5 and 10 kg/cm^2^) and VIPS exposure times (0 and 10 min) prior to direct non-solvent-induced phase separation (NIPS). Surface wettability analysis revealed that the optimized 50 wt.% activated carbon configurations were superhydrophilic (0° water contact angle), exhibiting instantaneous fluid absorption driven by strong capillary forces within the highly hygroscopic matrix. Morphological and gravimetric evaluations demonstrated that minimizing compaction (5 kg/cm^2^) and bypassing VIPS generated large macrovoids, resulting in the highest bulk internal porosity (61.05%) and maximum continuous gravity-driven water flux. Conversely, incorporating a 10-min VIPS exposure shifted the internal structure toward an interconnected sponge-like network. This structural transformation yielded the highest bovine serum albumin (BSA) rejection rate (12.97%) when paired with low pressure, as the network extended fluid residence time and maximized exposure to the activated carbon adsorption sites. Applying high compaction pressure (10 kg/cm^2^) to VIPS-treated membranes induced excessive polymer encapsulation of the active particles, significantly reducing separation efficiency while concurrently maximizing initial uniaxial tensile strength. Ultimately, these findings establish a foundational and highly tunable framework, demonstrating that calibrating mechanical compression alongside phase inversion dynamics balances permeability, adsorptive selectivity, and inter-particle binding cohesion for composite block membranes.

## 1. Introduction

Activated carbon (AC) is a highly efficient, cost-effective, and widely accessible adsorption medium utilized across various advanced separation applications, ranging from industrial wastewater treatment to biomedical filtration processes such as hemodialysis. Its robust adsorption capacity is driven by a vast internal surface area and strong Van der Waals forces, which facilitate the direct binding of target molecules, including macromolecules and proteinaceous pollutants, to its active surface [1,2]. Despite these favorable properties, the application of AC in its unbound, standalone form presents a critical structural vulnerability known as the “free particle hazard.” During continuous fluid flow, unbound carbon particles can easily dislodge and migrate into the treated stream. This particle leaching fundamentally compromises the purity and safety of the permeate, representing a severe operational risk in sensitive applications such as blood-contacting hemodialysis [3,4].

To effectively mitigate this hazard, it is imperative to securely immobilize the AC particles within a robust structural framework. Polymeric membranes, particularly polyethersulfone (PES), offer an excellent binder matrix due to their mechanical strength, inherent chemical stability, and established compatibility with stringent filtration processes [5]. Consolidating AC with a PES binder into composite blocks or discs creates a porous architecture that combines the size-exclusion selectivity of the polymer with the adsorptive capacity of the carbon [6]. Similar hybrid membrane approaches have recently demonstrated high efficacy in advanced separation applications, such as the selective discrimination of ions using functionalized metal–organic framework matrices [7]. In membrane filtration research, bovine serum albumin (BSA) is frequently employed as a standard model protein to evaluate the separation efficiency of these composites, owing to its well-characterized physicochemical properties and its relevance as a reliable analogue for target macromolecules in hemodialysis and other separation studies [8].

While the integration of AC into a PES matrix resolves the particle leaching issue, current fabrication methodologies present notable operational limitations. Polymeric composites frequently encounter a fundamental trade-off between achieving high selectivity and maintaining adequate permeability [9,10]. Previous studies on AC-PES membrane blocks have demonstrated limited water flux, primarily attributed to the application of excessively high compaction pressures during the initial fabrication stages [11]. Furthermore, composites fabricated solely via conventional non-solvent-induced phase separation (NIPS) tend to develop a thick, dense polymeric skin layer. This dense layer severely encapsulates the embedded AC particles [12], forcing permeating fluids to bypass the carbon’s active surface area. This structural masking fundamentally restricts the interaction time between the permeate and the adsorbent, compromising overall separation efficiency.

Overcoming these challenges requires a multifaceted modification of the fabrication process. Reducing the applied compaction pressure offers a direct pathway to improve water flux while preserving the composite’s structural integrity. Concurrently, integrating vapor-induced phase separation (VIPS) prior to the NIPS method presents a strategic approach to mitigate the formation of the dense skin layer. This dual-phase separation mechanism is hypothesized to create open microstructural pathways, allowing the permeate unimpeded access to the active adsorption sites of the AC.

Therefore, the primary objective of this study is to systematically analyze the concurrent effects of VIPS-NIPS exposure time and compaction pressure on the performance of AC-PES composite discs. By evaluating these parameters concerning their impact on structural morphology, permeability, and BSA protein rejection, this research aims to determine the optimal operational efficiency of the modified membrane system. Ultimately, optimizing these fabrication variables is intended to maximize the capability of the composite discs for targeted macromolecule removal, advancing their potential for rigorous filtration applications such as hemodialysis.

## 2. Materials and Methods

### 2.1. Materials

Polyethersulfone (PES, 5900P, Sumitomo Chemical Co., Tokyo, Japan) was utilized as the primary polymer matrix, with activated carbon powder (50 mesh, 297 µm) incorporated as the functional adsorbent filler. Polyvinylpyrrolidone (PVP-K90, PT BASF Indonesia) was employed as a pore-forming agent to enhance the hydrophilicity of the composite. N-methyl-2-pyrrolidone (NMP, Merck & Co., Inc., Darmstadt, Germany) served as the solvent, and distilled water functioned as the non-solvent during the phase inversion process. For filtration performance evaluations, bovine serum albumin (BSA, HiMedia Laboratories Pvt. Ltd., Thane, India) and phosphate-buffered saline (PBS, pH 7.4) were used as the model protein and buffer solution, respectively.

### 2.2. Fabrication Methods

**Figure 1:**
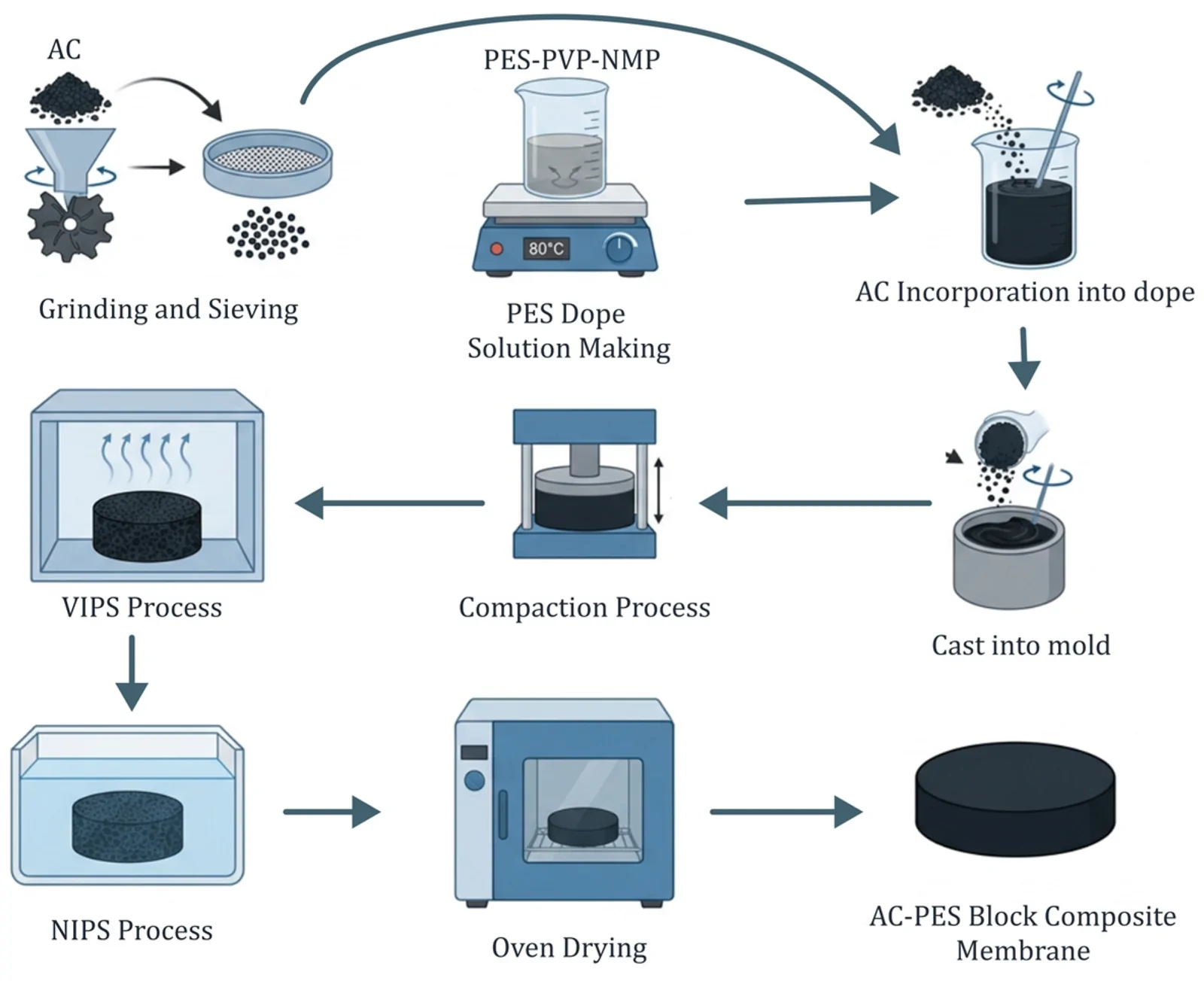
Schematic illustration of the fabrication process for the AC-PES block composite membrane. The workflow details the initial grinding and sieving of activated carbon (AC), the preparation of the PES-PVP-NMP polymeric dope solution at 80 °C, and the subsequent incorporation of AC. The mixture is then cast into a mold and subjected to mechanical compaction, followed by sequential phase inversion via the vapor-induced phase separation (VIPS) and non-solvent-induced phase separation (NIPS) processes. The procedure concludes with oven drying to yield the final composite matrix.

The fabrication process for the polyethersulfone (PES)-activated carbon composite disc membrane is schematically illustrated in Figure 1. Initially, activated carbon fragments were mechanically ground and sieved to isolate granules sized between 50 and 100 mesh, which served as the filler phase. Concurrently, the polymer matrix was prepared by dissolving a 20 wt.% polymer mixture (comprising PES and the PVP pore-forming agent) in 80 wt.% N-methyl-2-pyrrolidone (NMP). This solution was continuously stirred at 80 °C until optically clear and homogeneous.

To form the composite, the sieved activated carbon was gradually incorporated into the viscous polymer solution under continuous manual stirring to prevent particle agglomeration. The resulting homogeneous paste was then cast into a custom mold (40 mm in diameter). Following initial manual levelling to minimize void formation, the composite was compacted into a solid cylindrical structure using a hydraulic press.

The molded composite subsequently underwent a sequential phase inversion process to develop its porous architecture. First, vapor-induced phase separation (VIPS) was applied by exposing the membrane to a controlled humidity chamber (90–99% RH) to induce surface porosity within the polymer binder. This was immediately followed by non-solvent-induced phase separation (NIPS) via immersion in a water coagulation bath for 12 h to fully solidify the internal support structure. Finally, the resulting composite membrane was dried in an oven at 100 °C to evaporate any residual water prior to characterization.

### 2.3. Experimental Design

Because the sole objective of this initial phase was to map the broad compositional limits and establish a standardized baseline formulation for the main study, these evaluations were conducted as single exploratory screening trials. Consequently, statistical replicates were not generated for this preliminary optimization phase.

Prior to the primary experimental phase, a preparatory framework was established to optimize the composite’s baseline formulation. First, the compositional ratio of activated carbon within the matrix was evaluated, as this ratio directly dictates the overall adsorption capacity and filtration performance of the resulting membrane [13]. Second, the concentration of PVP within the polyethersulfone dope solution was optimized prior to fabrication. Controlling the PVP concentration is critical, as this additive significantly influences the morphological structure and pore distribution throughout the polymeric binder phase [14]. By standardizing these fundamental compositional variables beforehand, the subsequent experimental trials could more accurately isolate and evaluate the distinct effects of the targeted fabrication parameters on the optimized composite material. 

#### Membrane Composition 

Optimizing the constituent ratio is a foundational requirement, as it directly dictates the operational performance of the composite membrane. The primary focus of this preliminary evaluation was the incorporation of AC, which functions simultaneously as a structural filler and an active adsorbent within the polymeric matrix. To systematically investigate this parameter, AC was mixed into the polyethersulfone (PES) dope solution at targeted loadings of 30 wt.%, 40 wt.%, and 50 wt.%. The resulting composite variants underwent material characterization, emphasizing morphological analysis and water contact angle measurements. In this context, the water contact angle serves as a critical metric because it reliably correlates with the anticipated water flux performance of the finalized composite structure [15]. Understanding these microstructural and hydrophilic changes at varying AC loadings is essential for maximizing the membrane’s ultimate filtration efficiency.

In conjunction with AC loading, the incorporation of PVP into the dope solution was evaluated due to its significant effect on the porosity of the polymer binder. The addition of PVP acts as a pore-forming agent, which enhances the overall permeability of the composite framework. However, this introduces a mechanical trade-off: an excessively porous structure inherently weakens the binding capability of the primary polymer matrix [16]. This reduction in binding performance can lead to structural deformation and premature physical failure of the composite membrane under operational stress. To optimize this balance, PVP concentrations were tested across a range of 0 wt.%, 2 wt.%, 3 wt.%, 6 wt.%, and 8 wt.%. Evaluating these specific intervals provided the empirical data necessary to identify the threshold where matrix porosity is maximized without compromising the structural integrity of the composite binder.

### 2.4. Morphological Characterization

Morphological analysis of the composite membranes was conducted to visually substantiate the functional performance data. The primary objective of this characterization was to compare the structural alterations induced by the combined VIPS-NIPS fabrication method against those produced by the conventional NIPS-only approach. Preliminary macroscopic evaluations utilized an optical microscope at 200× magnification. This initial assessment provided an overview of the surface topography and the macroscopic distribution of activated carbon particles within the polymeric matrix. Subsequently, to obtain a detailed understanding of the internal microstructural architecture, the synthesized membranes were subjected to scanning electron microscopy (SEM). High-resolution SEM was necessary to directly observe the formation or mitigation of the restrictive dense skin layer, as well as to verify the development of the interconnected porous network facilitated by the VIPS mechanism.

It should be noted that standard quantitative pore size distribution analyses, such as capillary flow porometry was not employed in this study. The composite membranes fabricated herein are thick, solid cylindrical blocks (40 mm in diameter) formed through mechanical compaction, rather than conventional polymeric thin films. This specific, highly compacted form factor limits the viability of standard gas or liquid displacement techniques, which frequently induce edge channeling or structural fracturing when applied to thick matrices under the required high testing pressures. Consequently, this study relies on high-resolution SEM for the qualitative evaluation of pore architecture and matrix topography, complemented by the gravimetric method to quantify the total macroscopic bulk void volume.

### 2.5. Measurement of Membrane Porosity

Porosity is a fundamental morphological parameter that dictates a membrane’s functionality, specifically its permeability and flux. Defined as the ratio of pore volume to the total membrane volume, higher porosity typically corresponds to greater fluid flux due to reduced flow resistance [17]. The primary objective of porosity evaluation was to determine the total bulk volumetric void space essentially the macro-level fluid retention capacity of the solid composite blocks. The gravimetric method directly measures this bulk property, providing a representative quantification that reliably correlates with the gravity-driven water flux performance observed during our permeability testing.

The final porosity of a membrane is primarily governed by the dope solution formulation and the specific conditions during phase inversion. While higher polymer concentrations generally yield a denser matrix with lower porosity, the influence of additives varies significantly. Hydrophilic pore-forming agents leach out during coagulation to create voids and increase porosity. Conversely, the incorporation of solid fillers such as activated carbon can elevate the solution viscosity, which may induce delayed demixing and slightly reduce the overall porosity of the composite.

The overall membrane porosity (ε) was determined utilizing the gravimetric method. Composite membrane samples of specified dimensions were immersed in deionized water for 24 h to ensure complete pore saturation. After removing excess surface moisture, the wet weight ($W_{w}$) was recorded. The samples were subsequently dried in a vacuum oven until a constant weight was achieved to obtain the dry weight ($W_{d}$). The porosity was then calculated using the following Equation (1) [18]:

(1) $$\varepsilon \left(\%\right)=\frac{W_{w}-W_{d}}{\rho_{H_{2}O\;}\times A\times L}\times 100\%$$

where $\rho_{H_{2}O\;}$ is the density of water, A is the membrane area, and L is the membrane thickness.

The primary objective of our porosity evaluation was to determine the total bulk volumetric void space essentially the macro-level fluid retention capacity of the solid composite blocks. The gravimetric method directly measures this bulk property, providing a representative quantification that reliably correlates with the gravity-driven water flux performance observed during our permeability testing.

### 2.6. Surface Wettability Evaluation

The water contact angle (WCA) serves as a metric to characterize a membrane’s surface wettability, specifically defining its degree of hydrophilicity or hydrophobicity at the solid–liquid–gas interface [19,20]. This parameter directly influences overall filtration efficiency; highly hydrophilic surfaces (WCA < 90°) facilitate greater water flux due to their strong water affinity and form a dense hydration layer that improves resistance against organic fouling. To quantify this property, WCA measurements were conducted utilizing the sessile drop technique (5 µL of pure water) on a dry membrane surface [21].

### 2.7. Water Permeability Analysis

**Figure 2:**
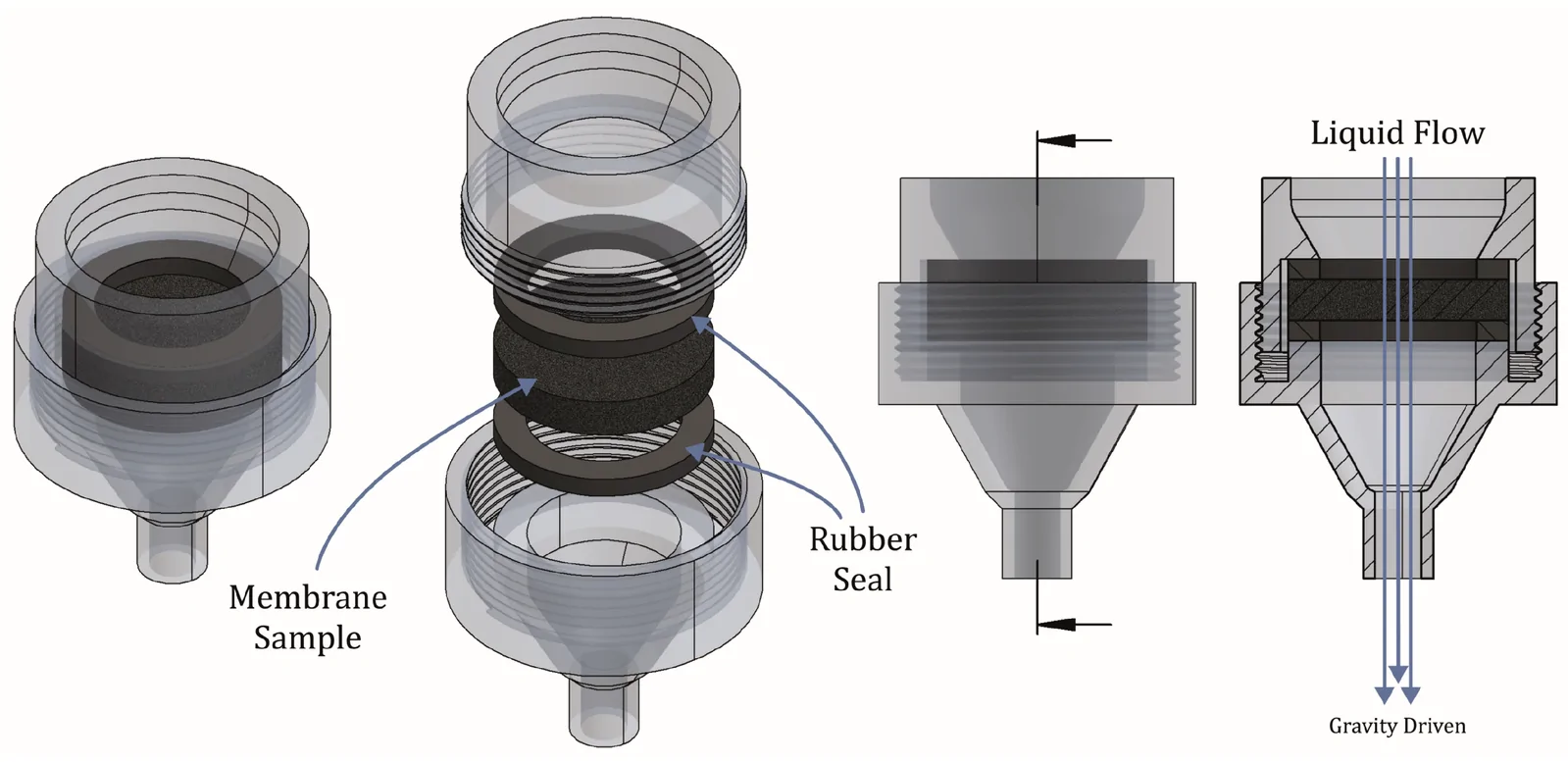
Schematic illustration of the custom-designed dead-end filtration module used for permeability and rejection evaluations. The exploded 3D view details the internal assembly, where the thick composite membrane sample is securely clamped between two rubber seals to prevent edge channeling and leakage. The cross-sectional diagram illustrates the gravity-driven filtration mechanism, ensuring unidirectional liquid flow directly through the porous composite matrix.

The productivity of the filtration system was evaluated by measuring its water flux, defined as the volumetric flow rate of fluid permeating through an effective surface area within a specific time frame. This quantitative metric serves as a direct indicator of the membrane’s structural and physicochemical properties; enhanced surface hydrophilicity and highly interconnected porosity significantly reduce flow resistance to maximize fluid transport. For the composite membrane fabricated in this study, permeability was evaluated utilizing a custom-built apparatus, as illustrated in Figure 2.

The procedure utilized a gravity-driven filtration mechanism, where a constant operating pressure was achieved by maintaining a stable liquid column of feed water above the membrane. To ensure steady-state flow, an initial stabilization phase was performed by allowing 10 mL of water to permeate prior to primary data collection. Following stabilization, the permeated water was collected over a 5-min interval and weighed using a digital balance. The resulting mass was subsequently converted to volumetric data utilizing the density of water. The final water flux ($J_{v}$) was calculated using the following Equation (2) [22]:

(2) $$J_{v}=\frac{Q}{A\times \Delta t}$$

where Q is the volume of permeate (L), A is the effective membrane area (m^2^), and Δt is the time interval (h).

### 2.8. Bovine Serum Albumin (BSA) Rejection Evaluation

Membrane selectivity, commonly referred to as rejection, evaluates the capacity of a membrane to retain specific solutes while permitting solvent passage, which directly determines the quality of the resulting permeate [23]. This separation capability is primarily governed by size exclusion mechanisms and physicochemical surface interactions. Generally, a narrower pore size distribution yields higher rejection rates for large macromolecules, albeit frequently at the expense of permeability [24,25].

To quantify this property, bovine serum albumin (BSA) was utilized as a standard model protein due to its established reliability in filtration studies. The characterization was conducted using the custom filtration apparatus previously employed for the permeability test. Following the continuous filtration of the feed solution, the BSA concentration in the permeate was analyzed using a UV–Vis spectrophotometer at a wavelength of 280 nm [26]. The optical absorbance values were converted to final protein concentrations utilizing a standard calibration curve. The overall rejection percentage (%SR) was subsequently calculated by comparing the solute concentration in the permeate against the initial feed solution concentration using the following Equation (3) [18]:

(3) $$\%SR=\left(1-\frac{C_{p}}{C_{f}}\right)$$

where $C_{p}$ is the concentration of the solute in the permeate and $C_{f}$ is the concentration of the solute in the initial feed solution.

### 2.9. Mechanical Property Characterization

The mechanical structural integrity of the composite membrane, which predominantly comprises an activated carbon filler bound by a Polyethersulfone (PES) matrix, is a critical parameter evaluated through tensile testing. This characterization is essential to quantify the binding strength of the polymer matrix and to ensure the membrane possesses adequate durability to withstand operational pressures in practical applications [27]. To determine this tensile strength, the membrane samples were evaluated using a motorized test stand. During the standardized procedure, the samples were securely clamped at a distance of 10 mm from each edge. A constant, controlled pulling speed of 5 mm/min was applied to observe the material’s gradual deformation response. The mechanical testing proceeded continuously until the sample reached its ultimate point of mechanical failure, marked by a complete fracture into two pieces at its peak tensile strength.

## 3. Results and Discussion

### 3.1. Optimization of Membrane Composition

#### 3.1.1. Optimization of Activated Carbon Loading

In this study, four composition variations in composite membranes were fabricated and evaluated. The four samples were differentiated based on the mass percentage of activated carbon relative to the total membrane mass, specifically 30%, 40%, and 50%, as presented in Table 1. To maintain comparative consistency, the composition of the binder polymer solution which constitutes the remaining mass balance for each sample—was standardized. This binder solution consisted of 18% Polyethersulfone (PES) as the primary polymer, 2% Polyvinylpyrrolidone (PVP) as the pore-forming agent, and 80% N-methyl-2-pyrrolidone (NMP) as the solvent. In addition to standardizing the solution composition, the fabrication parameters were also strictly controlled. These parameters included a vapor exposure time of 10 min during the vapor-induced phase separation (VIPS) process, and a compaction pressure of 5 kg/cm^2^ applied during disc formation.

**Figure 3:**
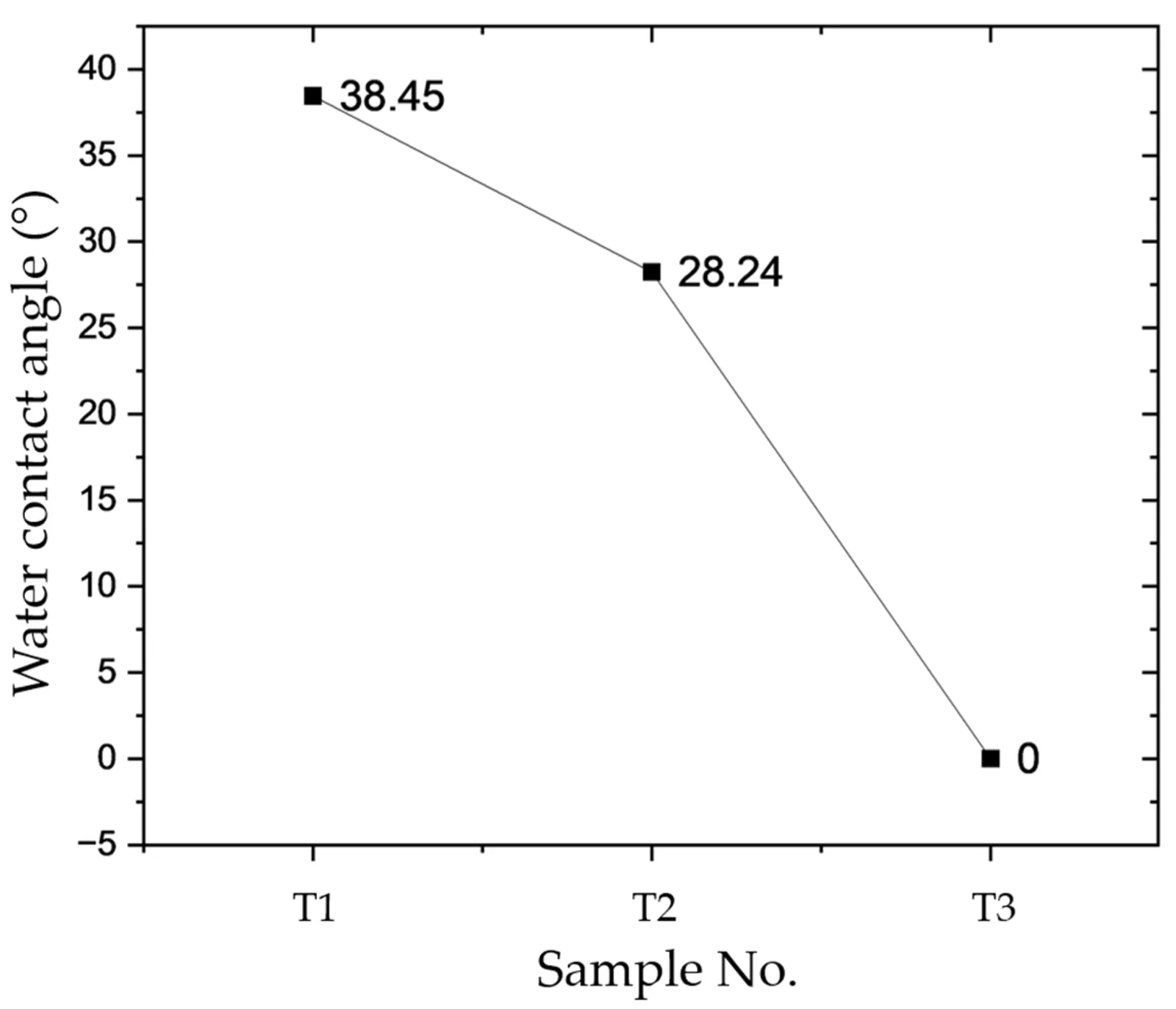
Surface wettability and dynamic water contact angle results across the evaluated membrane compositions. The measurements demonstrate the rapid absorption dynamics of the matrix, culminating in a superhydrophilic 0° contact angle driven by the incorporation of highly hygroscopic activated carbon.

The comprehensive WCA testing results for the three composition variations are illustrated within Figure 3. The acquired data from these tests revealed highly significant findings, wherein the absolute best characteristics were prominently discovered in the composite membrane containing 50 wt.% activated carbon. This specific sample successfully recorded an exceptional WCA value of 0°, which definitively classifies the membrane surface nature as superhydrophilic. The achievement of this superhydrophilic property is incredibly desirable for the final product because it directly correlates with a profoundly high permeability potential. Furthermore, achieving such an extreme degree of wettability ensures that the initial resistance against water permeation is completely minimized during filtration applications.

**Figure 4:**
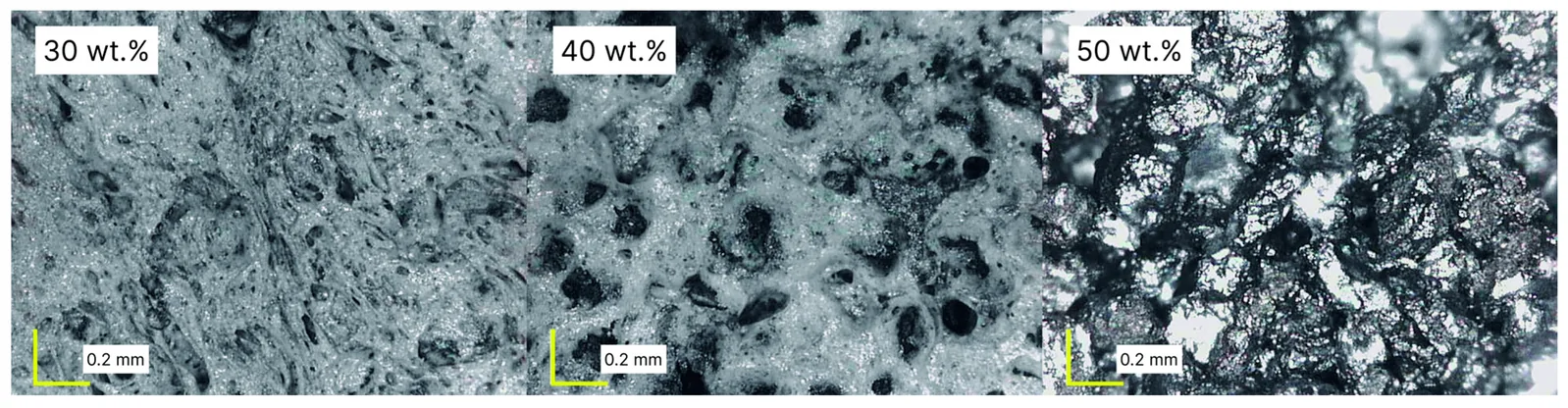
Morphological visual analysis of composite membranes at varying activated carbon (AC) compositions. At 30 wt.% AC, the membrane surface is heavily dominated by the PES matrix, leaving the AC particles largely obscured. At 40 wt.% AC, particles are more visible within the polymer-dominated structure. At 50 wt.% AC, well-distributed AC particles are uniformly coated by the PES layer without excessive masking, indicating a favorable particle-to-matrix interface that preserves access to the pore network.

These quantitative findings are corroborated by the morphological analysis in Figure 4. At 30 wt.% AC, the membrane surface was heavily dominated by the PES matrix, leaving the AC particles largely obscured. At 40 wt.% AC, the particles became more visible, though the structure remained polymer-dominated. However, at 50 wt.% AC, microscopic observations revealed well-distributed AC particles uniformly coated by the PES layer without excessive masking. This indicates a favorable particle-to-matrix interface that successfully preserves access to the activated carbon’s pore network.

Supported by the WCA results, the superhydrophilic 50 wt.% AC composition provided the optimal baseline, offering the highest permeability potential among the variations. Establishing this ideal ratio allowed the subsequent isolation and evaluation of mechanical fabrication variables, eliminating the confounding effects of compositional variance.

#### 3.1.2. Effect of PVP Concentration on Porosity and Mechanical

Polyvinylpyrrolidone is widely utilized as a pore-forming agent in membrane fabrication. However, its addition requires careful optimization, as excessive PVP concentrations can compromise the mechanical strength of the resulting composite structure [28]. To determine the optimal PVP composition, five composite membrane variations were prepared with PVP concentrations of 0, 2, 4, 6, and 8 wt.%. Initial characterization involved porosity testing to quantify the void volume within the solid membrane matrix.

**Figure 5:**
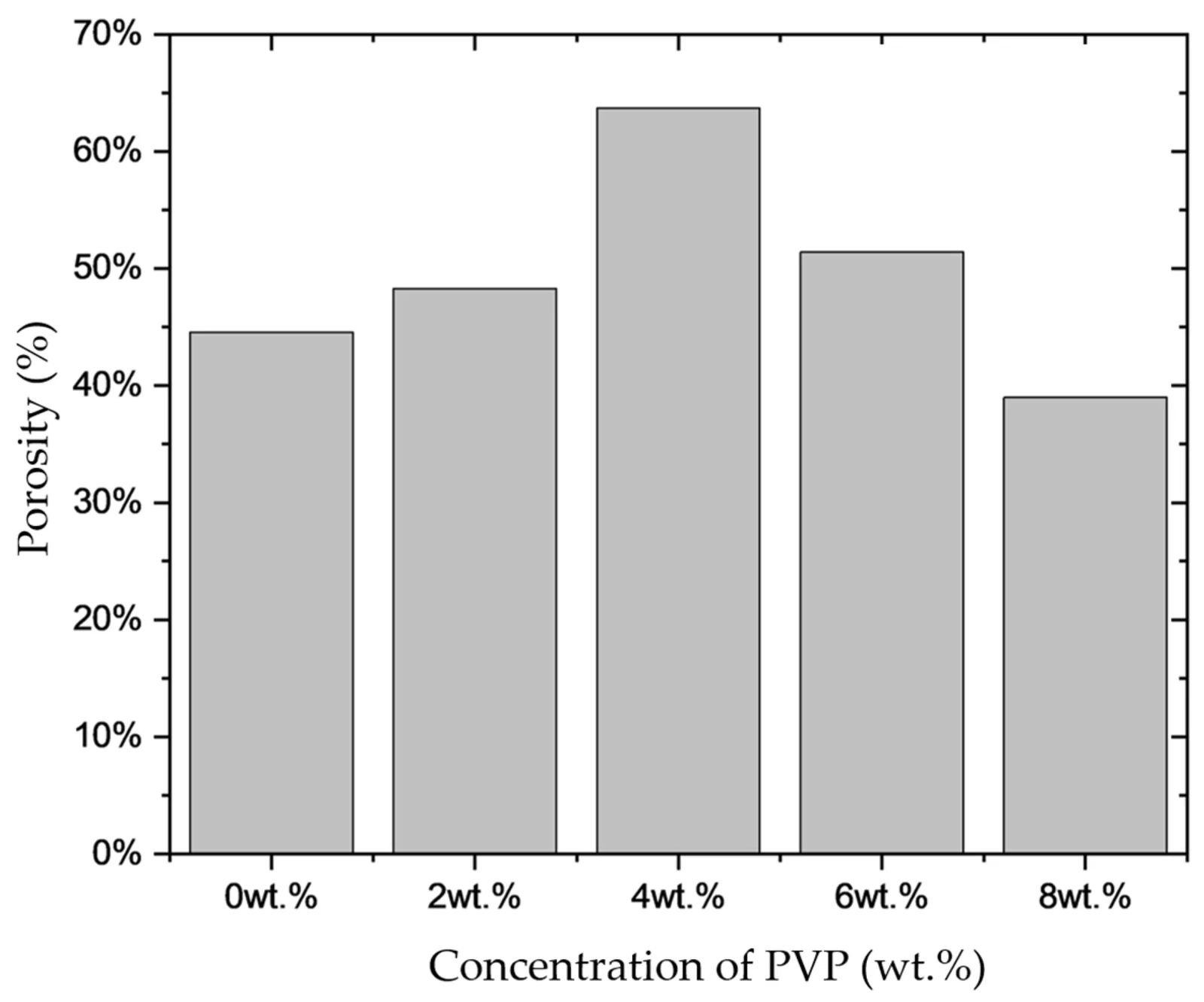
Effect of PVP concentration on membrane porosity. The void volume steadily increases with PVP addition up to an optimal concentration of 4 wt.% (yielding a maximum porosity of 63.69%), after which further addition results in a structural decline in overall porosity.

The porosity results for these variations, presented in Figure 5, indicate that porosity steadily increased with the progressive addition of PVP, reaching a maximum of 63.69% at a concentration of 4 wt.%. Conversely, further increases in PVP concentration to 6 wt.% and 8 wt.% resulted in a subsequent decline in overall porosity.

Subsequent evaluations involved water flux testing to assess the permeability performance of the membranes. During this phase, critical structural failures were observed in several samples, rendering them untestable. Specifically, composite membranes fabricated with 4, 6, and 8 wt.% PVP disintegrated immediately upon installation into the testing apparatus and initial exposure to water flow. Only the samples formulated with 0 wt.% and 2 wt.% PVP demonstrated adequate structural integrity to withstand the preparation and testing procedures.

**Figure 6:**
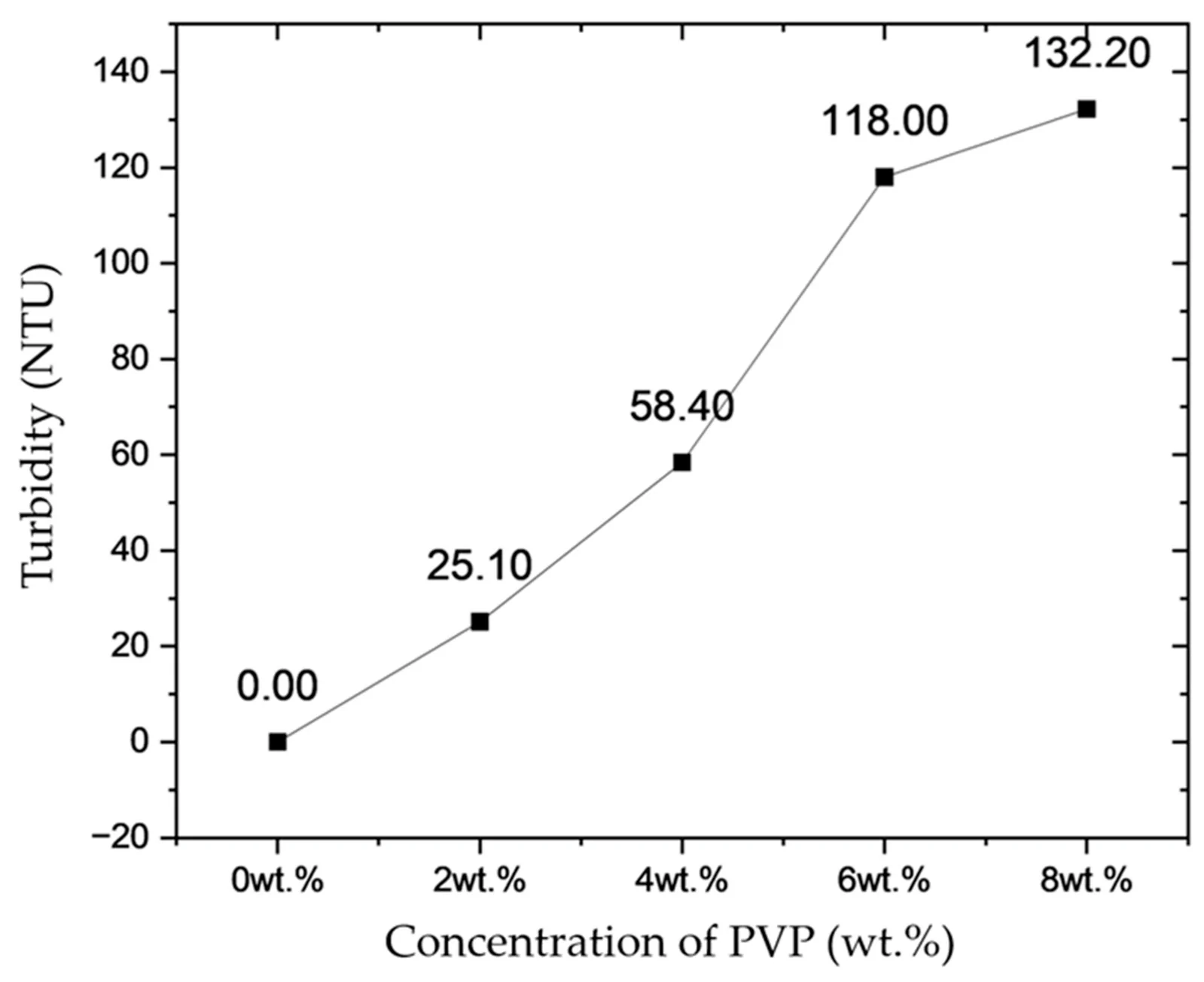
Turbidity values of the NIPS coagulation bath as a function of PVP concentration. The proportional increase in turbidity at higher concentrations (≥4 wt.%) indicates the excessive dispersion of activated carbon particles, corroborating the structural weakening observed in the composite matrix.

These mechanical failures were further corroborated by qualitative observations made during the fabrication process. It was noted that the coagulation bath used during the Non-Solvent-Induced Phase Separation (NIPS) stage exhibited increasing turbidity corresponding to the increased PVP concentration in the polymer solution. A turbidity test on the NIPS immersion water was conducted to validate this observation. The measurement results, depicted in Figure 6, confirmed that the turbidity of the coagulation bath increased proportionally with the percentage of added PVP.

Based on these two evaluations, it can be concluded that the addition of PVP at higher concentrations (≥4 wt.%) severely compromises the structural integrity of the composite membrane. This indicates that excessive PVP potentially weakens the interfacial bonding between the activated carbon particles and the PES polymer matrix. Evidence of this structural deterioration is demonstrated by two distinct phenomena: the physical disintegration of the membrane samples under light mechanical stress during the water flux test preparation, and the dispersion of activated carbon particles into the NIPS coagulation bath, which directly caused the elevated turbidity.

**Figure 7:**
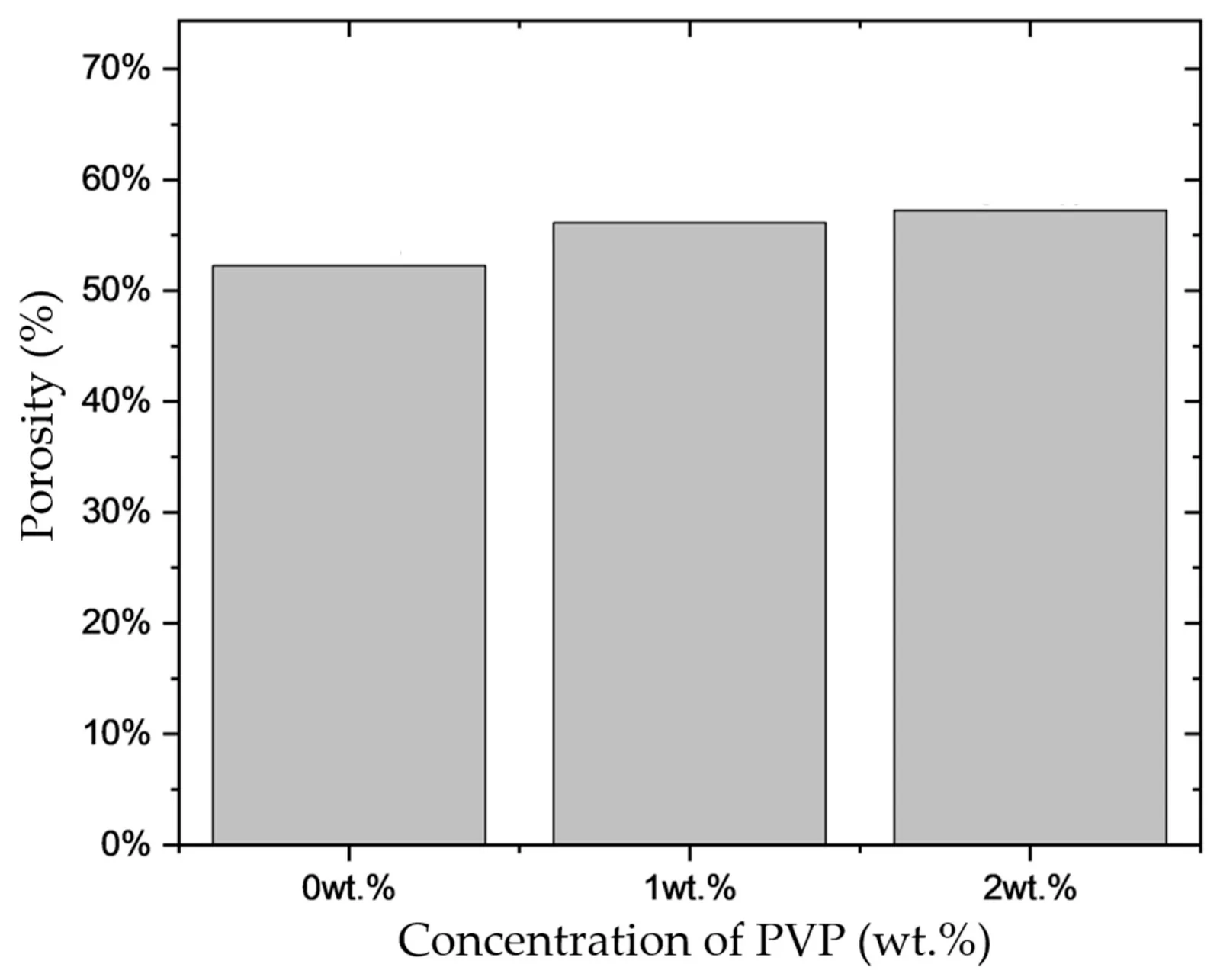
Porosity of composite membranes at the narrowed PVP concentration range. The data demonstrates a peak measured porosity of 57.22% for the sample containing 2 wt.% PVP.

Subsequent evaluations focused on the narrowed compositional range of 0, 1, and 2 wt.% PVP. The initial characterization conducted was a porosity test to evaluate the total void volume within the membrane structure. The complete results of these porosity measurements are graphically presented in Figure 7. Data analysis revealed that the highest porosity value was achieved by the sample formulated with 2 wt.% PVP, yielding a measured porosity of 57.22%.

**Figure 8:**
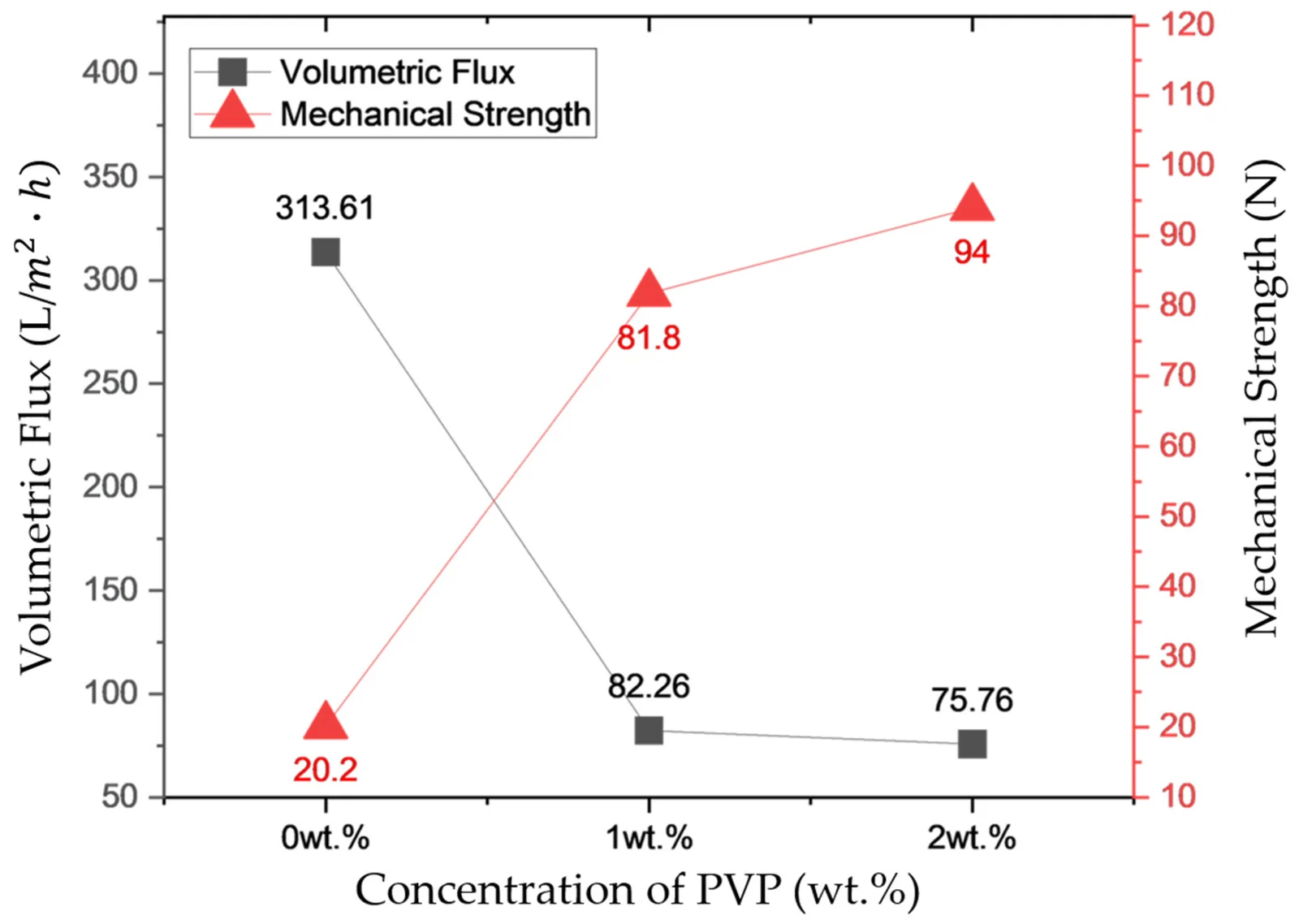
Permeability and mechanical evaluation of the composite membranes at the narrowed PVP concentration range. The data illustrates both the gravity-driven water flux and the maximum tensile load for each variation. While the 0 wt.% PVP sample recorded the highest permeability (313.605 L/m^2^.h), it suffered from critical structural disintegration. Conversely, the 2 wt.% PVP sample demonstrated superior mechanical integrity, withstanding a maximum tensile load of 94 N, confirming the necessity of PVP as a binding enhancer to secure the matrix.

The performance of the three samples was subsequently evaluated using a gravity-driven water flux test to measure permeability without external pressure. As shown in Figure 8, the 0 wt.% PVP sample exhibited the highest water flux (313.61 L/m^2^.h), while the 2 wt.% PVP sample exhibited the lowest (75.76 L/m^2^.h). However, post-test physical observations of the 0 wt.% PVP sample revealed structural disintegration at the edges, indicating insufficient interfacial bonding between the activated carbon particles within the matrix.

To address these structural failures, a tensile test was conducted to quantify mechanical strength (Figure 8). The results indicated that the 2 wt.% PVP sample possessed the highest inter-particle bonding strength, withstanding a maximum tensile load of 94 N before fracturing. This demonstrates that PES alone cannot provide sufficient bonding in this composite membrane, and PVP addition is required to secure the activated carbon particles.

Based on these characterizations, the 2 wt.% PVP composition was selected for the primary experimental phase. Despite yielding the highest water flux, the 0 wt.% PVP sample was discarded due to its mechanical unviability. The 2 wt.% PVP formulation was chosen because it achieves an optimal balance, providing superior mechanical integrity while sustaining adequate water flux performance for further study.

### 3.2. Determination of Fabrication Parameter Levels

Establishing the factorial levels for the fabrication parameters was a prerequisite for the primary experiments. Compaction pressure, the first factor, was set at 5 kg/cm^2^ and 10 kg/cm^2^. The second factor, VIPS exposure time, was evaluated at 0 and 10 min. A 10-min duration effectively induces pore formation when using an NMP [29], while a 0-min duration bypasses the VIPS stage entirely. In the latter case, compacted samples are immediately immersed in the coagulation bath to undergo only standard NIPS. This approach provides a baseline to directly compare conventional NIPS membranes against those fabricated via the combined VIPS/NIPS method. These established parameters and their factorial levels are summarized in Table 2.

### 3.3. Surface Morphology and Polymer Distribution

**Figure 9:**
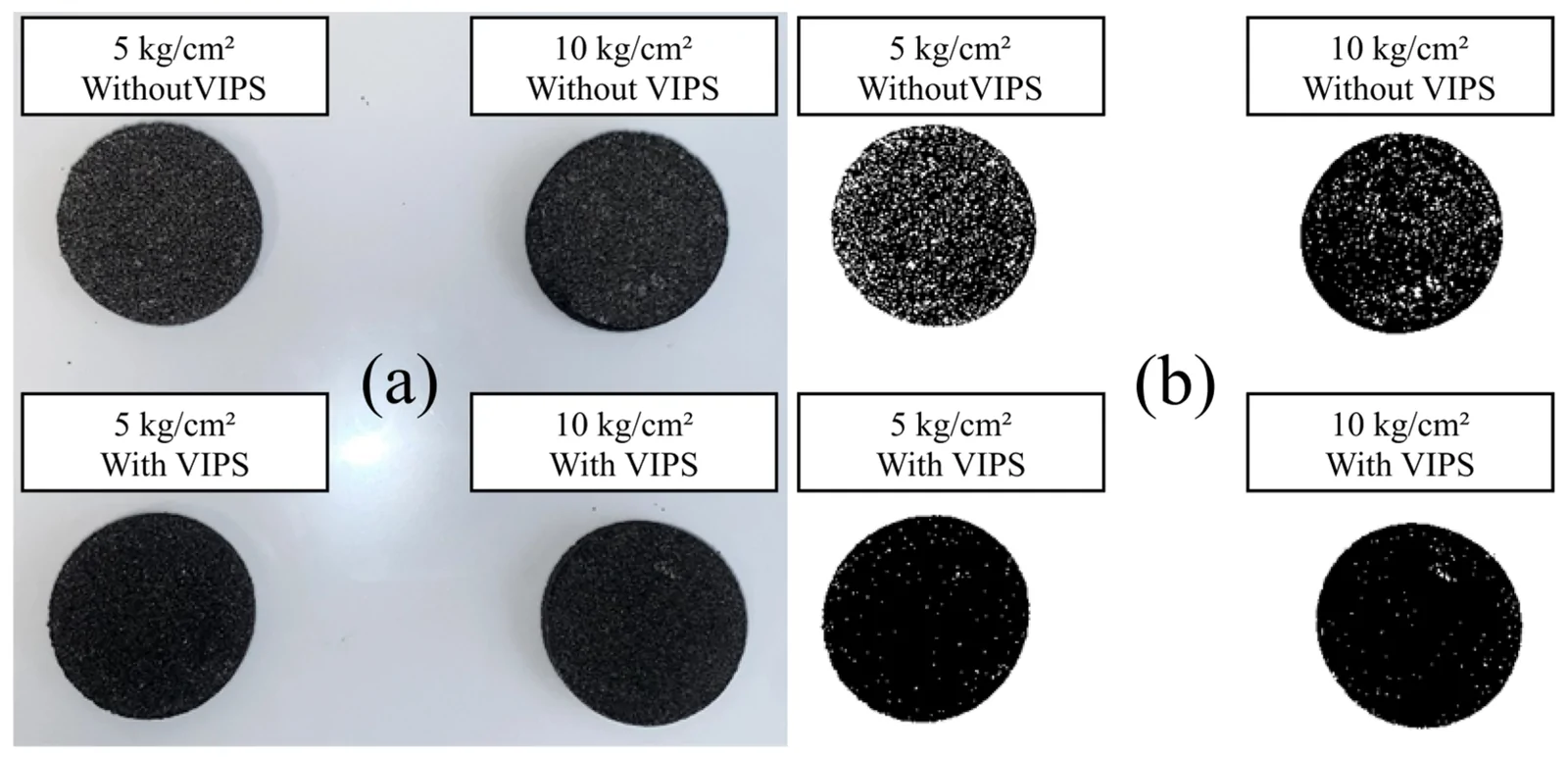
Macroscopic surface morphology of the fabricated composite membranes under varying compaction pressures and VIPS exposure times. (**a**) Standard optical photographs of the membrane surfaces and (**b**) their corresponding dithered binary transformations. The binary analysis highlights the surface distribution of the PES polymer binder (rendered in white) across the activated carbon matrix (rendered in black). The visual data indicates that fabrication without VIPS results in significant polymer surface coating, whereas the introduction of a 10-min VIPS treatment minimizes polymer masking, keeping the carbon particles largely exposed.

Morphological analysis was conducted to qualitatively evaluate the internal pore structure and surface characteristics of the composite membranes, utilizing standard photography and digital microscopy. As shown in Figure 9, macroscopic images were captured under centralized lighting to ensure consistency across samples. The visual structure is predominantly characterized by black activated carbon particles, with white speckles indicating the PES polymer binder on the particle surfaces.

To enhance this color-based visual analysis, the images were processed using a dithering method to create binary (black and white) representations (Figure 9). This transformation clearly highlighted structural differences, particularly regarding the fabrication method. Samples fabricated without VIPS displayed more prominent white speckling, indicating an extensive PES polymer coating on the activated carbon. In contrast, VIPS-fabricated samples were heavily dominated by black, suggesting minimal PES polymer accumulation on the particle surfaces.

**Figure 10:**
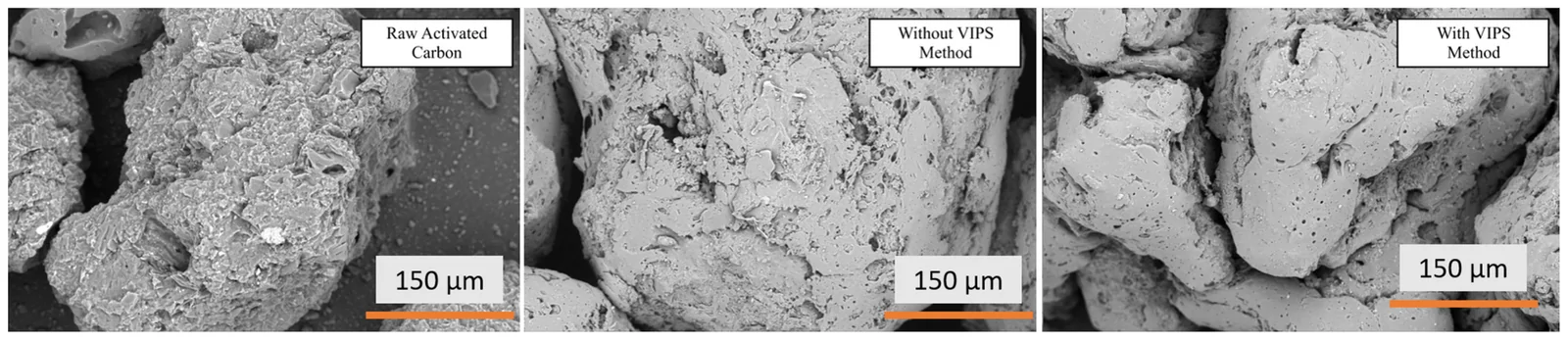
High-resolution SEM micrographs detailing the localized surface morphology of AC particles within the composite matrix. Compared to the smoothed, tightly encapsulated surface of particles fabricated without VIPS, the combined VIPS/NIPS method induces a refined micro-texture and the formation of visible macropores, successfully mitigating dense polymer masking.

Scanning electron microscopy was utilized to visualize localized surface changes on the AC particles at high resolution (Figure 10). While pristine AC particles inherently possess a rough, irregular topography, encapsulation by the PES polymer significantly altered this structure. Membranes fabricated without VIPS exhibited smoothed particle surfaces with limited visible porosity. Conversely, particles subjected to the combined VIPS/NIPS method demonstrated the distinct formation of newly generated macropores. Alongside this increased porosity, VIPS-treated samples displayed a refined micro-texture, contrasting sharply with the tightly encapsulated particles in non-VIPS samples. These findings confirm that integrating the VIPS method optimizes surface morphology by simultaneously inducing a porous network and reducing dense polymer masking.

As shown in Figure 10, the structural density and apparent clustering of the granules are intended consequences of the mechanical compaction process, which is essential for maintaining the block’s physical durability. However, as demonstrated by the VIPS-treated samples, this necessary compaction does not compromise functionality; the delayed phase inversion generates visible macropores and a refined micro-texture that keeps the tightly packed carbon particles exposed for optimal adsorption.

While these microscopic evaluations successfully confirm the structural transformations and macropore formation induced by the VIPS and NIPS processes, precise quantitative pore size distribution could not be definitively measured due to the macroscopic, compacted block nature of the composite matrix. Therefore, the overall structural evaluation relies on the synthesis of these qualitative SEM observations with the total bulk porosity measurements and functional BSA rejection indicators. Together, these metrics provide a comprehensive contextualization of the effective internal network tortuosity and the operational separation limits of the fabricated composite blocks.

### 3.4. Water Contact Angle and Absorption Dynamics

Water Contact Angle (WCA) measurement is essential for evaluating membrane wettability, a property that directly influences permeability. During testing, a 5 µL water droplet was dispensed onto the dry composite membranes. All tested samples exhibited a WCA of exactly 0°, classifying the surfaces as superhydrophilic. This exceptionally high-water affinity is a strong indicator of excellent permeability potential for water-based filtration.

**Figure 11:**
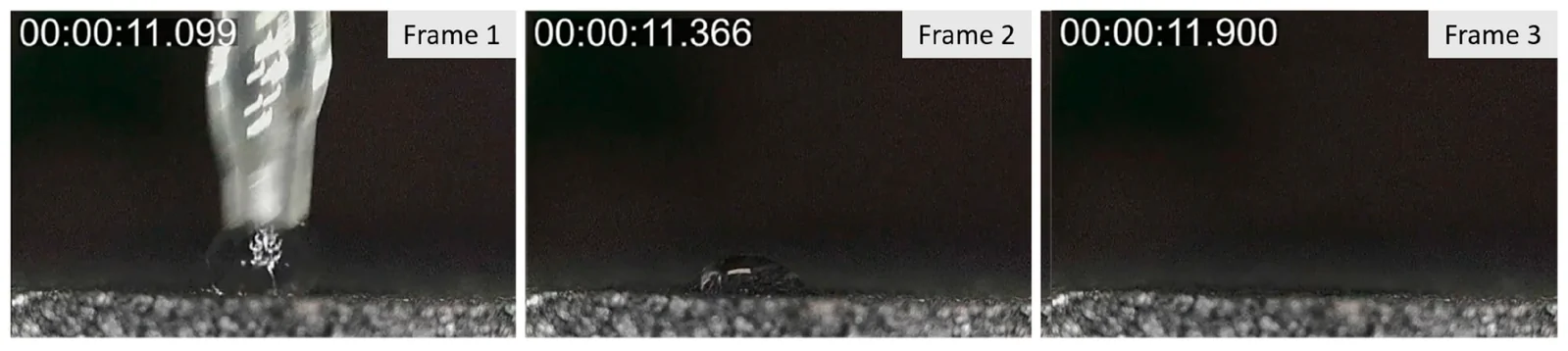
Time-lapse sequence illustrating the dynamic water absorption of the composite membrane. (**Frame 1**) Initial contact of the 5 µL water droplet at 11.099 s. (**Frame 2**) Rapid spreading and structural permeation at 11.366 s. (**Frame 3**) Complete absorption achieved at 11.900 s, resulting in a 0° water contact angle (WCA). The total wetting time of 0.801 s demonstrates the superhydrophilic surface characteristics driven by the highly hygroscopic activated carbon matrix. Note: Error bars are not applicable for samples with a 0° contact angle, as the instantaneous fluid absorption driven by the highly hygroscopic matrix prevents the measurement of steady-state angular variance.

This superhydrophilic nature is further supported by the visual absorption dynamics recorded in Figure 11. Upon initial surface contact at 11.099 s, the droplet rapidly spread (11.366 s) and was completely absorbed into the membrane matrix by 11.900 s. This equates to an instantaneous total absorption time of merely 0.801 s. Such highly effective, rapid water attraction is driven by the dominant presence and strongly hygroscopic nature of the activated carbon within the composite structure.

As previously established during the compositional optimization (Section 3.1.1), while lower activated carbon loadings yielded measurable contact angles (38.45° and 28.24° for 30 wt.% and 40 wt.% AC, respectively), the optimized 50 wt.% AC composition exhibited a water contact angle of exactly 0°. It is crucial to clarify that this universal 0° measurement across all primary experimental samples (M1–M4) does not merely indicate extreme surface spreading, but rather complete and instantaneous fluid absorption into the bulk matrix.

Because the composite is heavily loaded (50 wt.%) with highly hygroscopic activated carbon, the membrane acts similarly to a microscopic sponge. Upon contact, the immense internal surface area of the carbon network, combined with the macroscopic void structure, induces aggressive capillary action. As illustrated in the dynamic time-lapse sequence (Figure 11), the 5 µL droplet does not rest on the membrane surface; instead, it is rapidly wicked into the internal matrix, achieving complete absorption in exactly 0.801 s. Consequently, the testing instrument immediately records a 0° angle, which serves as a definitive indicator of the superhydrophilic and highly permeable nature of the optimized composite blocks.

### 3.5. Effect of Fabrication Parameters on Permeability

**Figure 12:**
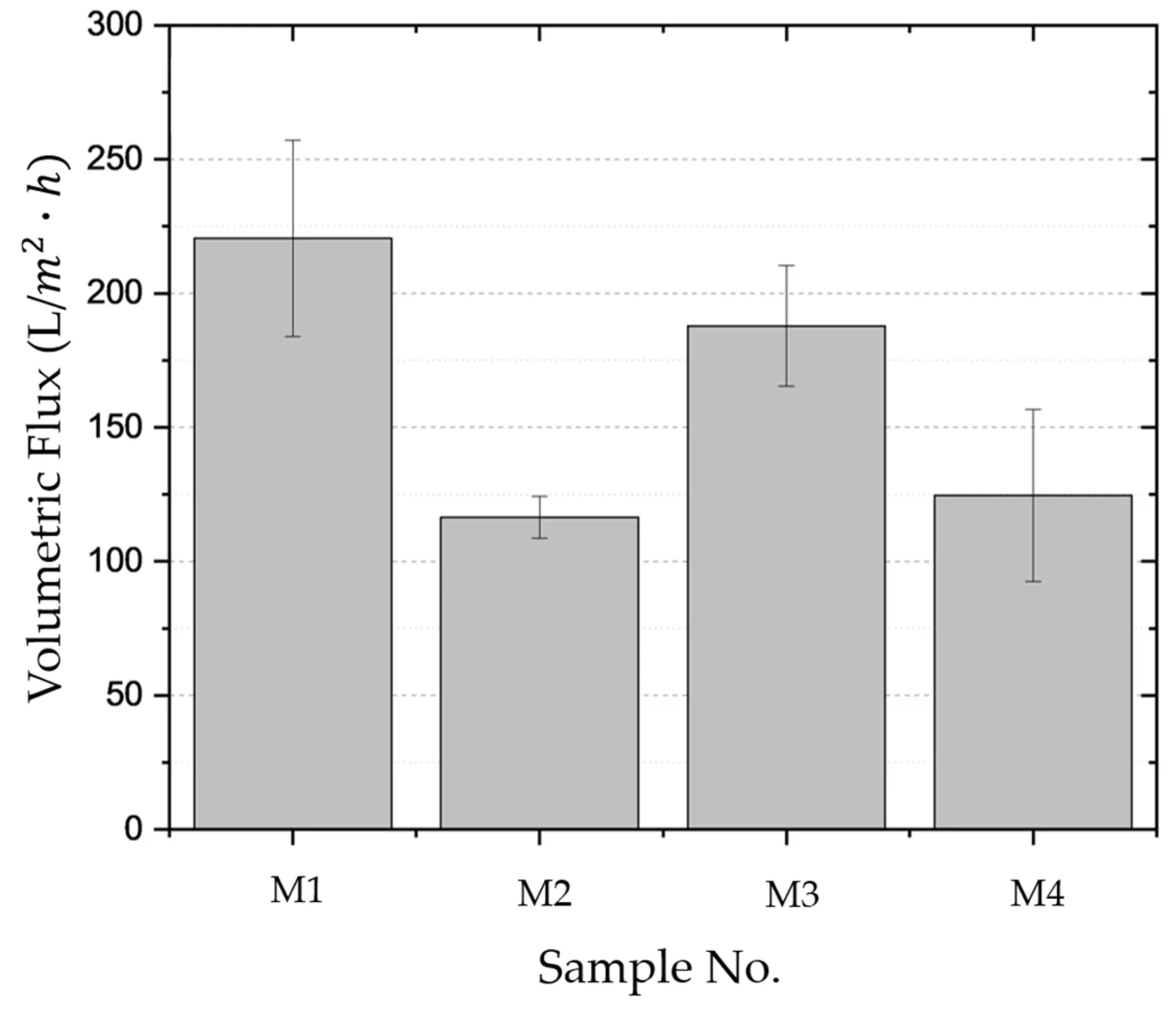
Water flux performance of the composite membranes under varying fabrication parameters. The maximum permeation rate was achieved at a compaction pressure of 5 kg/cm^2^ without VIPS exposure (0 min). Conversely, increased compaction pressure (10 kg/cm^2^) and the application of the VIPS method (10 min) both resulted in decreased water flux due to structural densification and increased pathway tortuosity.

Based on the fabrication parameters outlined in Table 3, the permeability characterization results presented in Figure 12 demonstrate that the sample compacted at 5 kg/cm^2^ without VIPS exposure (0 min) achieved the highest water flux. This maximum permeation rate aligns with the sample’s highly porous profile, indicating that minimizing external compressive forces and bypassing the delayed phase inversion process promotes rapid fluid transport through the composite matrix.

Analysis of the data reveals an inverse relationship between applied compaction pressure and water flux. Increasing the compaction pressure from 5 kg/cm^2^ to 10 kg/cm^2^ resulted in a consistent decline in the water permeation rate across all tested samples. This reduction in overall permeability is driven by the physical compression of the internal membrane matrix. The increased mechanical force tightly packs the activated carbon particles and the binding polymer, shrinking available micro-channels and constricting internal pore pathways. This structural densification introduces higher hydraulic resistance, ultimately limiting the total volumetric throughput capable of passing through the membrane structure.

Incorporating the VIPS method into the fabrication sequence exerted a similar restricting effect on overall fluid permeability. Composite membranes subjected to a 10-min vapor exposure consistently exhibited lower water flux values compared to samples formed solely through the direct immersion (NIPS) method. This decline in filtration performance is attributed to morphological transformations driven by the delayed phase inversion mechanism. Prolonged exposure to the vapor environment mitigates the spontaneous formation of highly permeable macroscopic voids, shifting the internal structure toward a denser, interconnected sponge-like network. While this cellular structure is beneficial for improving internal particle stability, it increases the tortuosity of the fluid pathways, thereby restricting the maximum achievable water flux of the finalized composite.

### 3.6. Effect of Fabrication Parameters on Porosity

**Figure 13:**
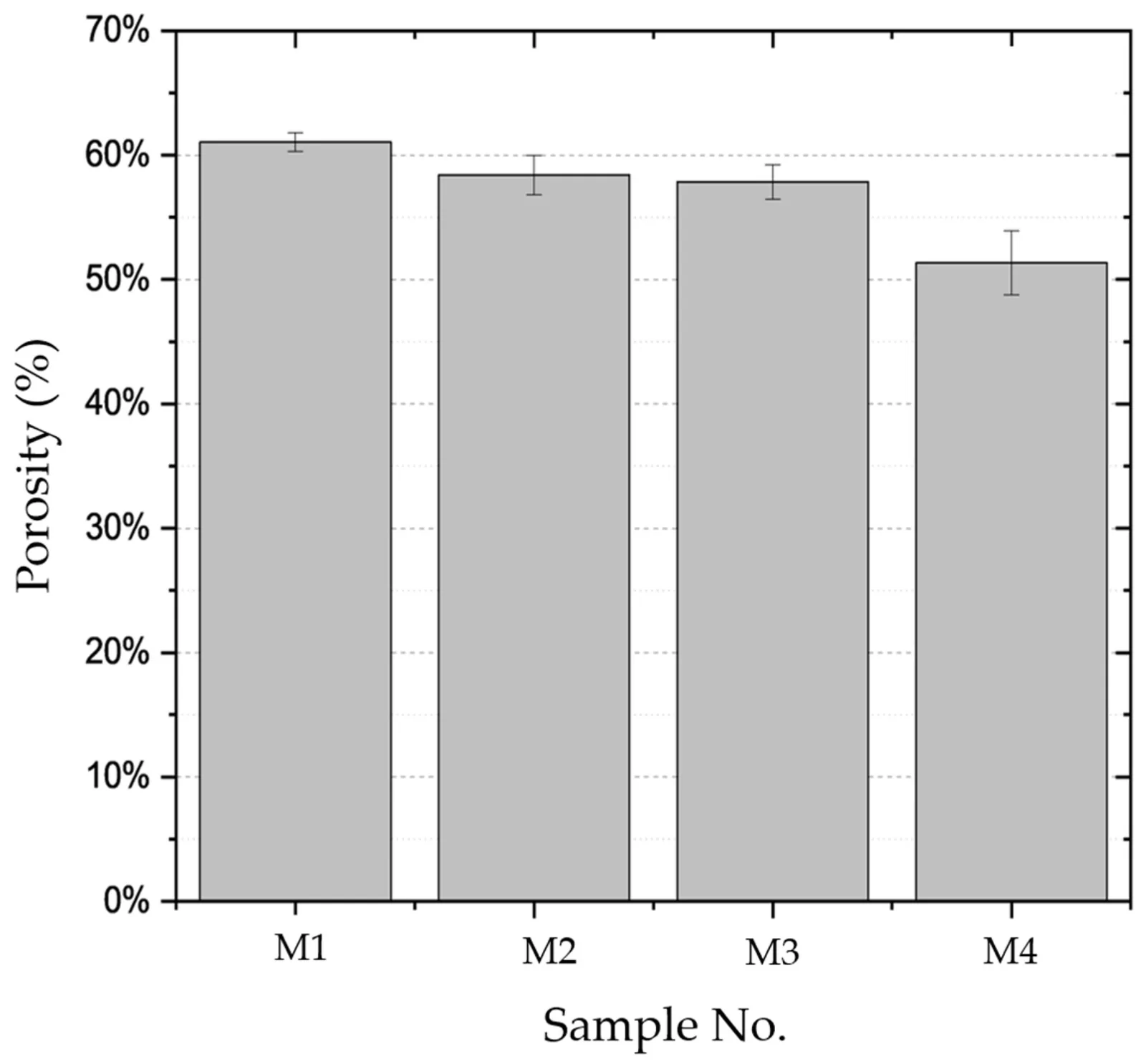
Porosity characterization results of the fabricated composite membranes. The data illustrates that increasing compaction pressure and incorporating VIPS exposure both result in a denser matrix, with the maximum porosity (61.05%) observed at the lowest fabrication settings (5 kg/cm^2^ and 0 min VIPS).

Porosity evaluation results, detailed in Figure 13, demonstrate that both compaction pressure and vapor exposure time significantly dictate the void volume of the membrane matrix. The maximum average porosity (61.05%) was achieved using the lowest parameter settings: a 5 kg/cm^2^ compaction pressure without VIPS exposure (0 min). This indicates that mitigating external compression and bypassing delayed phase inversion maximizes internal void retention. The experimental data highlight an inverse relationship between compaction pressure and porosity. Increasing pressure from 5 kg/cm^2^ to 10 kg/cm^2^ consistently re-duced total porosity across all samples. This reduction is driven by physical compaction mechanics [30], where elevated pressure tightly packs the activated carbon particles and PES matrix together, shrinking interstitial spaces and creating a denser structural profile. Similarly, incorporating the VIPS method restricted overall porosity. Samples subjected to 10 minutes of vapor exposure exhibited lower porosity than those formed via direct NIPS. While NIPS promotes the rapid formation of large, finger-like macrovoids, VIPS delays the demixing process, encouraging a denser, finely intercon-nected sponge-like structure [31]. Consequently, although VIPS produces a highly uniform morphology, it suppresses macrovoid formation, thereby reducing the total volumetric porosity of the final composite.

### 3.7. Effect of Fabrication Parameters on Rejection Performance

**Figure 14:**
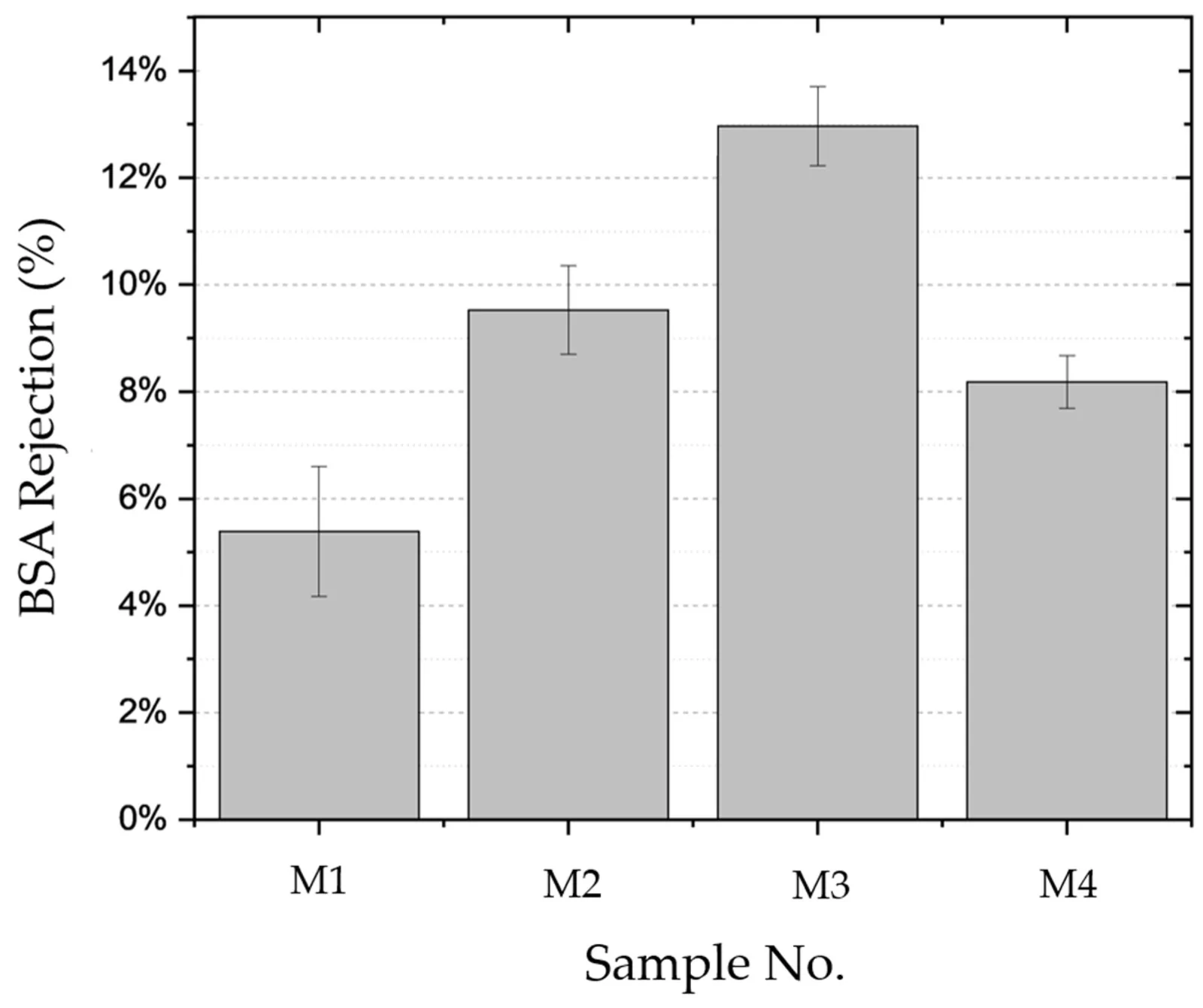
BSA rejection characterization results of the fabricated composite membranes. The maximum rejection rate of 12.97% is achieved by combining low compaction pressure (5 kg/cm^2^) with a 10-min VIPS exposure.

The protein rejection capabilities of the composite membranes are presented in Figure 14. The maximum average rejection rate (12.97%) was achieved by the sample fabricated with a 5 kg/cm^2^ compaction pressure and a 10-min VIPS exposure. This indicates that combining lower compressive forces with delayed vapor exposure creates an optimal internal matrix for target protein capture.

While a 12.97% BSA rejection rate may appear low compared to traditional ultrafiltration membranes designed strictly for size exclusion, this figure must be evaluated in the context of the membrane’s primary design objective: balancing active site exposure with high-throughput permeability. Unlike conventional polymeric matrices that rely on narrow pore channels to physically sieve macromolecules, this AC-PES composite utilizes an adsorption-dominated separation mechanism.

This enhanced filtration is driven by a sponge-like pore structure formed during the delayed phase inversion of the VIPS process. This interconnected network allows the BSA solution to penetrate the matrix rather than being completely blocked by a dense surface skin layer. By bypassing this restrictive skin layer, the open microstructural pathways increase internal fluid residence time, facilitating the effective physical adsorption of dissolved proteins onto the activated carbon particles via Van der Waals forces across their extensive surface area [32].

**Figure 15:**
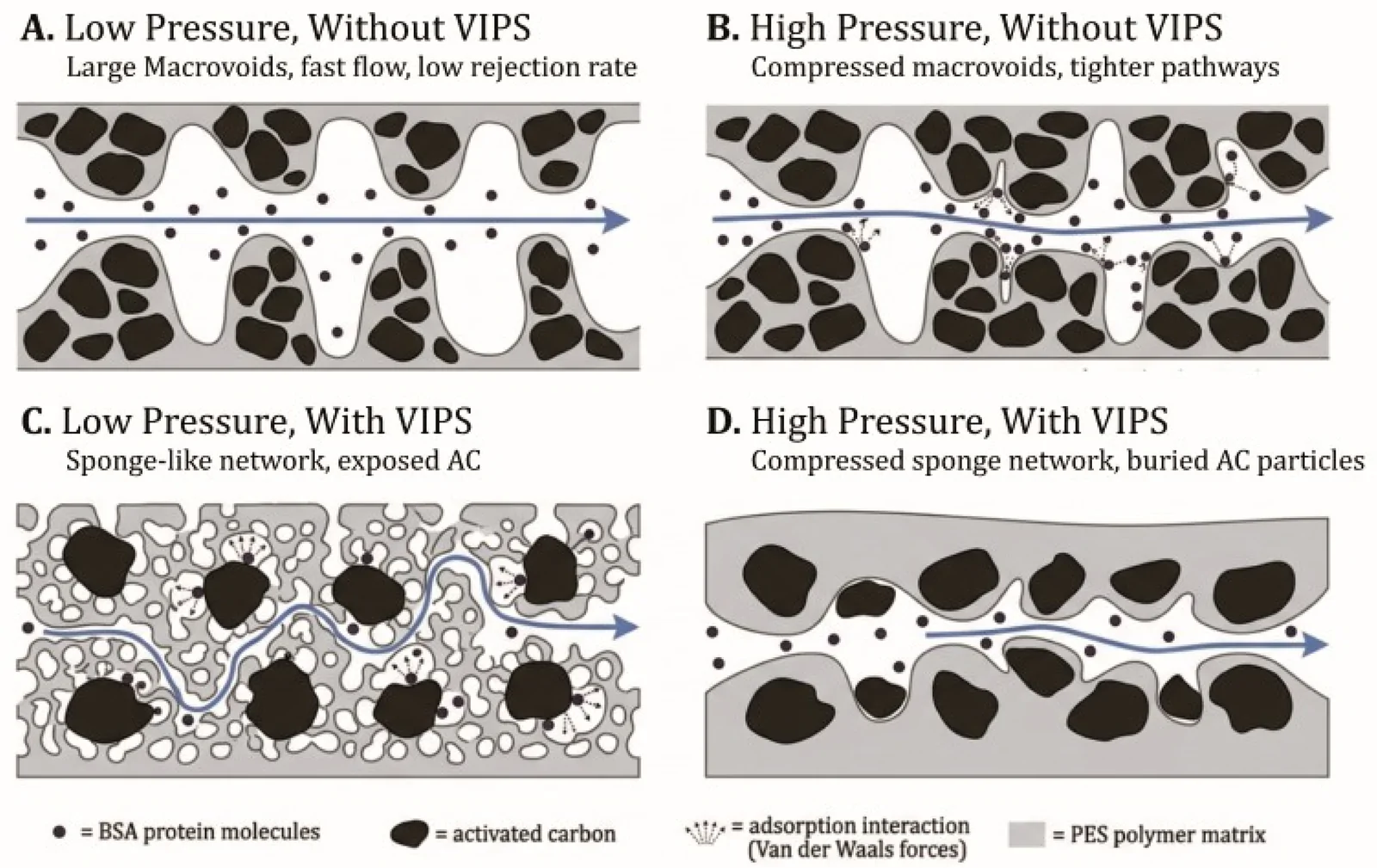
Schematic illustration of the internal membrane morphology and its effect on BSA protein filtration mechanisms under varying fabrication parameters. (**A**) Low pressure (5 kg/cm^2^) without VIPS yields large macrovoids, resulting in fast fluid flow and a low rejection rate. (**B**) Applying high pressure (10 kg/cm^2^) without VIPS compresses these macrovoids, creating tighter pathways that enhance mechanical protein trapping. (**C**) Combining low pressure with VIPS generates an interconnected sponge-like network with exposed activated carbon (AC) particles, maximizing fluid tortuosity and Van der Waals adsorption interactions for the highest rejection rate. (**D**) Conversely, applying high pressure with VIPS excessively compresses the sponge network, burying the AC particles within the PES polymer matrix and blocking critical adsorption sites.

A complex interplay exists between compaction pressure and VIPS exposure, as modeled in Figure 15. Without VIPS (0 min), increasing pressure from 5 kg/cm^2^ to 10 kg/cm^2^ improved rejection (from 5.39% to 9.53%) by shrinking internal macrovoids and enhancing target capture via size exclusion. However, with VIPS applied (10 min), increasing the pressure drastically reduced rejection (from 12.97% to 8.18%). This decline occurs because over-compressing the already dense VIPS-induced matrix causes the PES binder to excessively encapsulate the activated carbon particles. This polymeric encapsulation blocks protein access to critical internal adsorption sites, ultimately masking the carbon’s active surface and lowering the overall separation efficiency.

Consequently, the 12.97% rejection rate represents a calibrated operational threshold. Calibrating a lower compaction pressure (5 kg/cm^2^) alongside VIPS exposure preserves open pathways to the embedded adsorbent particles. This successfully mitigates the severe structural encapsulation common in conventional NIPS composites, achieving functional macromolecular retention without compromising continuous fluid permeation.

### 3.8. Effect of Fabrication Parameters on Mechanical Strength

**Figure 16:**
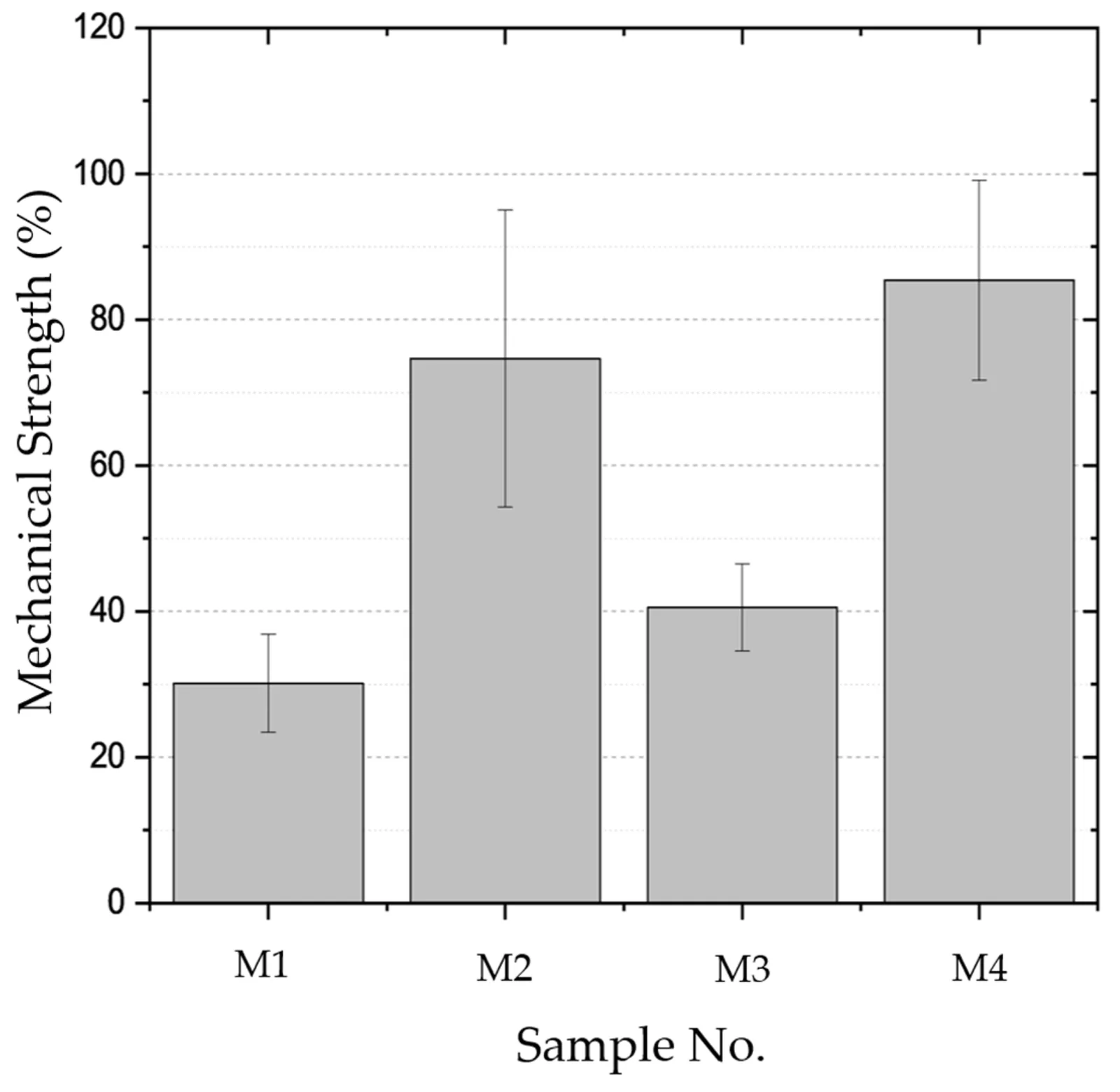
Mechanical strength characterization results of the fabricated composite membranes. The data illustrates a positive correlation between applied compaction pressure and physical durability, with the maximum tensile strength achieved under high compression (10 kg/cm^2^) and delayed phase inversion (10 min VIPS).

Evaluated through tensile testing, mechanical strength is a critical parameter dictating a composite membrane’s durability and practical operational lifespan. The tensile results, presented in Figure 16, demonstrate that fabrication parameters significantly influenced the ultimate tensile strength of the matrix. The maximum mechanical resilience was achieved using a 10 kg/cm^2^ compaction pressure and a 10-min VIPS exposure. This peak strength underscores a standard fabrication trade-off: the parameters that limit fluid permeability simultaneously enhance the matrix’s structural integrity.

The experimental data reveals a distinct positive correlation between compaction pressure and mechanical durability. Increasing the external pressure from 5 kg/cm^2^ to 10 kg/cm^2^ consistently enhanced the tensile strength across all samples. This improvement stems directly from the densification of the internal matrix during mechanical pressing. Higher compression firmly packs the activated carbon filler and the PES binder together, eliminating microscopic gaps and consolidating the materials. This resulting dense structural configuration minimizes internal voids, allowing the membrane to withstand significantly higher mechanical stress before physical rupture.

### 3.9. Comparative Performance Analysis with Similar Studies

To contextualize the performance and structural novelty of the fabricated AC-PES block composite membranes, a comprehensive comparison with similar filled polymeric membranes in recent literature was conducted (Table 4). Traditional non-solvent-induced phase inversion methods for fabricating PES ultrafiltration membranes incorporated with activated carbon frequently face severe permeability–selectivity trade-offs. While conventional PES/AC membranes can achieve increased pure water flux and enhanced hydrophilicity, they are typically limited to thin-film configurations rather than high-capacity block matrices [5].

Furthermore, surface modifications intended to reduce fouling in PES matrices—such as the incorporation of APTMS-modified activated carbon [33] or thiolated chitosan/AC composites [34] often result in unintended side effects. For instance, while modified AC can increase water flux through electrostatic repulsion, this same repulsive mechanism significantly decreases the adsorption and rejection of target proteins like BSA. Similarly, while hydrophilic composite fillers improve initial flux, these conventional flat-sheet membranes remain highly susceptible to gradual pore blocking and fouling under continuous pressure.

In contrast, the approach demonstrated in this study balances these competing parameters by calibrating mechanical compression alongside dual phase inversion dynamics (VIPS-NIPS). By introducing a 10-min VIPS exposure prior to NIPS immersion, the rapid formation of macrovoids is controlled, transitioning the internal matrix into an interconnected sponge-like network. When paired with a low compaction pressure (5 kg/cm^2^), this cellular framework prevents the PES binder from fully encapsulating the active particles. Consequently, the permeating fluid experiences extended internal residence time and direct contact with exposed AC adsorption sites, yielding an optimal BSA rejection rate of 12.97% driven by Van der Waals forces, while sustaining excellent gravity-driven water flux without requiring external operating pressure.

## 4. Conclusions

This foundational study investigated the synergistic effects of compaction pressure and Vapor-Induced Phase Separation (VIPS) on the initial structural and filtration performance of activated carbon/polyethersulfone (AC-PES) composite block membranes. The experimental and morphological analyses reveal a highly tunable matrix characterized by specific performance trade-offs:

1. Driven by the dominant presence of highly hygroscopic activated carbon, the optimized membrane matrix achieved a superhydrophilic 0° water contact angle, demonstrating rapid fluid permeation with a droplet absorption time of just 0.801 s.
2. Bypassing VIPS and applying minimal compaction pressure (5 kg/cm^2^) promoted the formation of large internal macrovoids. This configuration achieved the highest overall porosity (61.05%) and the maximum continuous water flux.
3. The highest BSA rejection rate (12.97%) was attained by combining low compaction pressure (5 kg/cm^2^) with a 10-min VIPS exposure. The delayed phase inversion generated an interconnected sponge-like network that extended internal fluid residence time while effectively exposing activated carbon adsorption sites.
4. Increasing compaction pressure to 10 kg/cm^2^ on VIPS-treated membranes physically consolidated the matrix, yielding the highest mechanical tensile strength. However, this densification proved detrimental to filtration selectivity, as it caused the polyethersulfone (PES) binder to excessively encapsulate the activated carbon, effectively blocking target protein access to essential adsorption sites.

Overall, this research demonstrates that carefully calibrating external compressive forces alongside phase inversion dynamics provides a highly tunable framework to balance permeability, adsorptive selectivity, and inter-particle cohesion. This allows the structural and operational profile of the AC-PES composite membrane to be precisely tuned for high-throughput permeability, optimal selectivity, or enhanced structural durability based on target application requirements. While this study successfully establishes an optimized baseline and highlights the viability of this specific fabrication method, we recognize that it represents a foundational assessment. Further research is essential to validate the operational longevity of these composites under real-world conditions. Therefore, comprehensive long-term operational evaluations specifically focusing on dynamic fouling resistance, permeability recovery, compressive stress tolerance, and extended mechanical stability under continuous cross-flow operation remain critical future milestones before the composite can be validated for rigorous industrial or biomedical applications.

## Figures and Tables

**Table 1:** Composition and fabrication parameters of the composite membranes.

Sample	Activated Carbon (wt.% of Total Mass)	PES (wt.% of Binder) *	PVP (wt.% of Binder) *	NMP (wt.% of Binder) *	Vapor Exposure Time (min)	Compaction Pressure (kg/cm^2^)
T1	30	18	2	80	10	5
T2	40	18	2	80	10	5
T3	50	18	2	80	10	5

* Note: The binder solution (comprising PES, PVP, and NMP) accounts for the remaining mass balance of the total membrane composition. For example, in Sample 1, the binder solution constitutes 70 wt.% of the total mass.

**Table 2:** Fabrication parameters and their evaluated values.

Fabrication Parameter	Unit	Evaluated Values
Compaction Pressure	kg/cm^2^	5, 10
Vapor Exposure Time (VIPS)	minutes	0, 10

**Table 3:** Summary of fabrication parameters and their corresponding effects on membrane structure and water flux.

Sample No.	Compaction Pressure	VIPS Exposure Time	Methodology
M1	5 kg/cm^2^	-	NIPS
M2	10 kg/cm^2^	-	NIPS
M3	5 kg/cm^2^	10 min	VIPS + NIPS
M4	10 kg/cm^2^	10 min	VIPS + NIPS

**Table 4:** Performance and structural comparison of the optimized AC-PES composite block against similar membrane studies.

Membrane Type/Matrix	Fabrication Method	Key Characteristics	Permeability & Rejection Performance	Structural Limitations/Advantages	Source
AC-PES Block Composite	Compaction (5 kg/cm^2^) + VIPS (10 min) + NIPS	Superhydrophilic (0° WCA); Instantaneous absorption; Exposed AC particles in sponge network.	High gravity-driven flux; 12.97% BSA Rejection via extended residence time.	Binds carbon particles securely to eliminate particle leaching while maintaining high flux in a solid block format.	This Work
PES/AC Composite UF Membrane	Conventional phase inversion with up to 1.5 wt.% AC filler	Enhanced hydrophilicity	Nearly doubled pure water flux at 2 bars compared to pristine PES; maintains BSA rejection capabilities.	Limited to thin-film ultrafiltration applications; highly reliant on external operating pressure.	[5]
PES/Modified-AC Antifouling Membrane	Phase inversion with 3-Aminopropyltrimthoxysilane (APTMS) modified AC	Highly porous with electrostatic repulsive surface properties.	Higher initial flux, but lower BSA rejection.	The electrostatic repulsion that prevents fouling actively decreases target protein adsorption efficiency.	[33]
Thiolated Chitosan/AC PES Composite	Phase inversion with multi-component hydrophilic fillers	Improved hydrophilic character (contact angle reduced to 52.2°).	Increased pure water flux.	Remains susceptible to standard pore blocking and flux reduction over time.	[34]

## Data Availability

The original contributions presented in the study are included in the article, further inquiries can be directed to the corresponding author.
